# Ecological river health assessments, based on fish ordination analysis of ecological indicator entities and the biological integrity metrics, responding to the chemical water pollution

**DOI:** 10.1007/s11356-024-32862-5

**Published:** 2024-03-27

**Authors:** Namsrai Jargal, Jeong-Eun Kim, Bilguun Ariunbold, Kwang-Guk An

**Affiliations:** 1 Department of Bioscience and Biotechnology, Chungnam National University, Daejeon, 34134 Republic of Korea; 2 Ecological Research Division, Korea National Park Research Institute, Wonju, 26411 Republic of Korea

**Keywords:** Wadable systems, River health, Fish, Community attributes, Ordination scores, Chemical stressors

## Abstract

Evaluation of the ecological health of rivers requires a focused examination of how biological indicators respond to chemical stressors to offer key insights for effective conservation strategies. We examined the influence of stressors on aquatic ecosystems by analyzing various ecological entities and biotic integrity metrics of fish communities. A nonmetric multidimensional scaling (NMDS) approach was applied to determine scores based on 19 fish ecological entities (FEs) and a fish-based multi-metric index of biotic integrity (mIBI-F). The composition of fish communities in reference clusters differed from the disturbed clusters due to instream chemical stressors. These chemical stressors, including high levels of nutrients, organic matter, and ionic/suspended solids, were linked to variation in the key indicator FEs, whose guild identities were closely associated with instream chemical degradation. The scores of FEs (abundance weighted) and mIBI-F metrics in the first NMDS axis (NMDS1) were significantly linked with chemical health indicators (*p* < 0.001), such as total phosphorus (*R*^2^ = 0.67 and 0.47), electrical conductivity (*R*^2^ = 0.59 and 0.49), and chlorophyll-*a* (*R*^2^ = 0.48 and 0.25). These NMDS1 scores showed better accuracy than the conventional mIBI-F score in capturing river ecological health linked with chemical health status as determined by a multi-metric index of water pollution. Our study suggests that based on the ordination approach, the biological integrity of these systems reflected the chemical health.

**Supplementary Information:**

The online version contains supplementary material available at 10.1007/s11356-024-32862-5.

## Introduction

Maintaining good water quality in rivers and streams is essential for their ecological health and ecosystem service provision (Carr and Neary 2008; Keeler et al. 2012). Chemical and biological evaluations are necessary to comprehensively assess river health for human use and aquatic conservation. Biological assessments, such as fish community analyses, can provide valuable insights into the effects of pollutants, habitat changes, and restoration efforts (Ibáñez et al. 2010; Bylak et al. 2022; Vadas et al. 2022). This study analyzed the ecological health of rivers through statistical scoring of fish community distributions in relation to chemical health indicators.

Assessing the health of river ecosystems based solely on chemical water quality variables provides an incomplete picture. Rapid and unpredictable changes in river hydrology make this difficult. Complementing such assessments with biological evaluations is essential to understand the ecological conditions that impact aquatic biodiversity (Barbour et al. 1999; Li et al. 2010). This is because the biological components of river ecosystems, such as fish and macroinvertebrates, are sensitive indicators of water quality and habitat conditions. Their presence, abundance, and diversity can reveal current and persistent impacts of pollutants, habitat changes, and stressors, offering important insights into the overall health of these water bodies (Li et al. 2010; Vadas et al. 2022; Bylak et al. 2024). By integrating biological assessments with chemical analyses, we can obtain a more holistic perspective of the complex dynamics of river and stream health, thereby enabling more informed and effective conservation and management strategies (Muñoz and Sabater 2014; Atique and An 2018).

In recent decades, a growing trend in biological assessment has been a focus on the ecological roles of species and community functional structures within ecosystems (Li et al. 2010; Oliveira et al. 2012; Yadamsuren et al. 2020). For instance, species clusters defined by the combination of their multiple traits are used as a base to calculate functional diversity and model aquatic community shifts driven by environmental factors and stressors (Mouillot et al. 2014; Chua et al. 2019; Wang et al. 2019; Nasi et al. 2023). In addition, analyses based on ecological guilds are a well-known method for assessing the ecological health of rivers and streams (Noble et al. 2007; Jargal et al. 2023). Compositional variation in specific guilds, such as trophic or tolerance guilds, can indicate the ecological condition of the systems in response to changes in instream water chemistry or riparian land use along the river (Gao et al. 2015; Mamun and An 2022). Habitat guild-based differences are locally associated with physical habitat conditions, such as meso-microhabitat diversity, flow regimes, and stream order (McCabe 2011; Spurgeon et al. 2019). However, guild-based analysis or assessment based only on one identity, such as the abundance of sensitive or tolerant species, is insufficient to capture ecological diversity due to natural variability among species (Verdonschot and van der Lee 2020; Jargal et al. 2022a). Therefore, combining species with identical guild identities or traits into a cluster (hereafter an ecological entity) will help capture the nuances of ecological diversity and the dominance of trait combinations within communities. Statistical modeling based on ecological entities would help determine variation in aquatic communities due to spatial changes in environmental factors and stressors, thereby supporting diagnoses of river health.

The multi-metric index of biotic integrity (mIBI) is a widely adopted tool for assessing the ecological health of aquatic communities and their responses to environmental stressors (Ibáñez et al. 2010; Vadas et al. 2022). Metrics selected for the mIBI commonly represent biological attributes, including species richness, composition, and individual health status. These metrics are well-established in their responsiveness to environmental stressors across various countries (Hering et al. 2006; Choi et al. 2011). Proposed initially by Karr (1981), the discrete scoring method has been widely used to assign initial scores to these metrics. However, more recent studies by Hering et al. (2006) and Stoddard et al. (2008) have advocated continuously scored metrics in mIBI assessment. The final mIBI score is calculated by combining and averaging individual metric scores, providing an assessment of ecological health status, categorized as “good,” “fair,” or “poor.” However, evaluating ecological conditions requires assessing how changes in the original values of indicators are associated with the quality of instream conditions and scores estimated by these values rather than the conventional scorings. To achieve this, using a matrix of site-specific values of mIBI metrics to apply multivariate ordinations would provide statistically reliable site scores based on the relations of metrics to environmental stressor gradients, thus improving the ecological assessment of river systems.

Fish play a crucial role in maintaining the health and stability of aquatic ecosystems (Simon and Evans 2017; Villéger et al. 2017). They are sensitive to changes in water quality and habitat conditions, which makes them useful indicators for assessing river health (Karr 1981; Pont et al. 2006; Jargal et al. 2023). Water quality deterioration driven by increased nutrient levels and organic matter often triggers declines in ecosystem specialists while fostering an increase in generalist species (Mamun and An 2022; Jargal et al. 2023). Certain fish species or groups, such as sensitive or tolerant species, also serve as indicators of the ecological health of river systems in response to changes in instream water chemistry and riparian land use (Chalar et al. 2013; Gao et al. 2015; Whitney et al. 2019). Thus, changes in fish community composition are a good diagnostic of ecological conditions by indicating functional shifts in aquatic systems under environmental stressors (Larentis et al. 2021; Gao et al. 2015; Jargal et al. 2022b). Developing ordination analysis based on fish community attributes can support ecological assessment of water quality by increasing statistical rigor while being biologically relevant.

Our study aimed to determine whether the nonmetric ordination-based scores of fish ecological entities (FEs) and fish-based mIBI (mIBI-F) metrics could effectively assess the ecological health status of rivers. We analyzed the nonmetric ordination-based scores of FEs and mIBI-F metrics in relation to chemical health indicators at 41 study sites. Furthermore, we examined the relationships between these ordination scores and spatial changes in elevation, stream order, riparian land use proportion, and chemical health scores assessed using a multi-metric index of water pollution (mWPI Score). Employing FEs and mIBI-F metrics, we suggest an ordination-based approach to scoring systems that can provide valuable insights into the ecological impacts of pollutants and habitat changes in rivers and streams. This approach will help us better delineate the ecological health of river ecosystems by capturing the nuances of ecological diversity and the dominance of trait combinations within fish communities.

## Materials and methods

### Study area

**Fig. 1:**
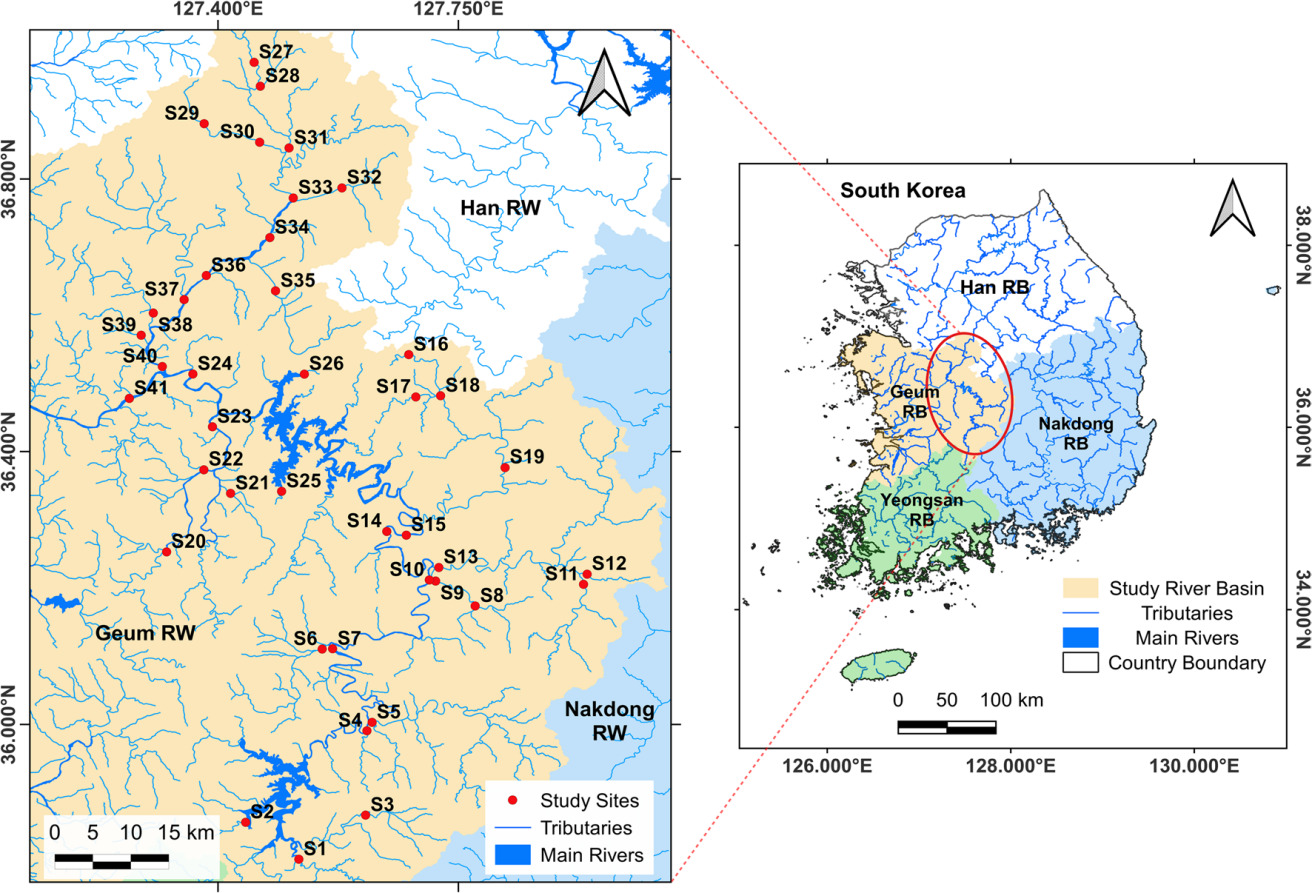
Map of the study region, the upper Geum River in South Korea, and the study site locations (*n* = 41)

The 41 study sites are located in the upper region of the Geum River Basin (Fig. 1). These sites represent the two main rivers, the Miho and Geum, along with their 19 tributaries. The reason for selecting the region was that using a lower spatial scale rather than a higher spatial scale, such as a river basin scale, is more effective for ecological assessments as it helps to minimize geographical variations (Cortes et al. 2013; Pompeu et al. 2023). Also, the spatial autocorrelation of sites was prudently considered to ensure each site was close to similar sites (Table S1). The study area is mostly covered by forests, with agricultural land, urban areas, and industrial development being the major land uses. The deterioration of water quality is primarily caused by nonpoint sources of pollution from urban and agricultural activities. These sources, along with some point sources such as wastewater treatment plants (S23 and S36), are mainly located in the Gap Stream (S21–S23) and Miho River (S34–S40) (Yang et al. 2021; Shiferaw et al. 2023). The impacts of these pollution sources are even more severe downstream at sites S24 and S41.

### Environmental data

Chemical water quality analysis was conducted using data from 12 chemical variables and sestonic chlorophyll-*a* (Chl-*a*). The chemical variables included water temperature (WT), dissolved oxygen (DO), electrical conductivity (EC), total suspended solids (TSS), total organic carbon (TOC), biological oxygen demand (BOD), total nitrogen (TN), ammonium-nitrogen (NH_4_-N), nitrate-nitrogen (NO_3_-N), total phosphorus (TP), orthophosphate (PO_4_-P), and TN:TP ratio. Data were collected in August and September 2016 as part of a national chemical monitoring program. These data were obtained from the Water Information Network System, maintained by the Ministry of Environment Korea (MOE), and accessible through http://water.nier.go.kr. The locations of the sampling sites are the same as the fish sampling sites. The elevation (Elev) of sites was defined using Google Earth Engine. Four variables were assessed for physical habitat variation, including stream order (SO) and land use proportion of agricultural (%Agr), urban (%Urb), and forest (%For) cover. A 500-m buffer circle was used to determine each site’s riparian land use proportion and the assessment was based on the Environmental Geographical Information System (https://egis.me.go.kr/main.do) managed by the MOE.

### Chemical health indicators

We used the mWPI to evaluate chemical health conditions (Kim and An 2015). The seven metrics of the mWPI represent four chemical water quality indicators: nutrient regime (TN, TP, TN:TP ratio), organic matter (BOD), suspended solids/ionic contents (TSS, EC), and primary productivity (Chl-*a*). Each metric was assessed by assigning a score of 5, 3, or 1 corresponding to concentration-based criteria for chemical water quality; total mWPI Scores were determined by summing the score for each metric. The score range is generally between 7 and 35, and a higher score indicates better chemical health in streams and rivers (Table S2).

### Fish sampling

Field sampling was conducted at study sites using cast and kick nets from mid-September to early October 2016, followed by the wading method (Barbour et al. 1999). Each collection lasted 40–50 min, during which all fish species were identified and any anomalies were recorded. Then these field observations were used to conduct a community-based analysis.

### Fish community attributes

Two fish community attributes were utilized to derive statistically significant site scores for assessing river health: FEs and the mIBI-F. The determination of FEs was based on the grouping approach proposed by Mouillot et al. (2014). An FE represents a cluster of fish species that exhibit an exact match in their ecological guild structures. We defined FEs based on ecological guilds of fish, including trophic, habitat, and tolerance guilds. There were three categories of trophic guild (TrG: insectivores [Ins], carnivores [Car], and omnivores [Omn]), four of habitat guild (HG: benthic [BT], riffle benthic [RB], riffle benthic and water column [RB-WC], and water column [WC]), and three of tolerance guild (TG: sensitive species [SS], intermediate species [IS], and tolerant species [TS]). The guilds were established using the regional identification guide for freshwater fishes of Korea (Han et al. 2015).

The eight metrics of the mIBI-F represent three indicators of fish community structure: species richness/composition, trophic composition, and fish abundance/individual health (An et al. 2006). These are listed in Table S3, including *M*_1_: total number of native fish species (NS), *M*_2_: number of riffle-benthic species (RB), *M*_3_: number of sensitive species (SS), *M*_4_: percentage of individuals belonging to tolerant species (%TS), *M*_5_: percentage of individuals belonging to omnivore species (%Omn), *M*_6_: percentage of individuals belonging to native insectivore species (%NIns), *M*_7_: total number of native individuals (NSI), and *M*_8_: percentage of individuals showing anomalies (%Ano). Scores were assigned and summed for a total mIBI-F Score, where a higher value indicates better biological health (Table S3).

### Statistical analysis

#### Hierarchical clustering

Cluster analysis was employed to hierarchically group distinct sites based on the dissimilarity (Euclidean distance) driven by chemical water quality variables. Before the analysis, a log transformation was done on the initial variables to equalize the influence of extreme values, stabilize variance, and obtain more interpretable clusters. The cluster dendrogram was built using the “*fviz_dend*” function in the “factorextra” package in conjunction with the hierarchical clustering method using “Ward.D2” performed in the R program (Ver. 4.2.2).

#### Nonmetric multidimensional scaling

We applied nonmetric multidimensional scaling (NMDS) ordinations of FEs and mIBI-F metrics to investigate changes in the fish community across study sites. Bray–Curtis distance was used to estimate the relative abundances of FEs and mIBI-F metric-defined variation among fish communities (study sites). The site scores of the NMDS ordinations were used to assess the health of streams in relation to the variation in chemical indicators and other environmental factors. The analysis was performed using the “*metaMDS*” function within the “Vegan” package of R (Oksanen et al. 2022). Two-dimensional scaling plots were built using the “*ordiplot*” function in the Vegan package of R.

#### Correlation and regression analysis

Pearson correlation was used to delineate the relative contribution of each FE and mIBI-F metric to the NMDS ordination. Simple linear regression was applied to determine the responses of fish communities to the variation in chemical health indicators. Log-transformed values of chemical health indicators were used in the analyses to meet normal distribution. The analyses were performed using SigmaPlot (Ver. 14.5, Systat Software Inc.), and regression plots were built using the “ggplot2” package in R. In addition, correlation analysis was conducted on the potential influences of Elev, SO, land use changes (%Agr, %Urb, and %For), and mWPI Score on the ordination scores of FEs and mIBI-F metrics, mIBI-F Score, and chemical health indicators. The correlation plot was built using the “ggcorrplot” package in R.

## Results

### Measured distribution of environmental variables across study sites

There was a good deal of variation in all environmental variables (Table 1), with the results suggesting potential degradation of chemical health. The mWPI Score ranged from 9 to 35, with a mean of 24, and the chemical health of the study sites was broken down as follows: 36.6% were excellent, 14.6% were good, 19.5% were fair, 19.5% were poor, and 9.8% were very poor (Table S2).

**Table 1:** A summary of variables representing chemical water quality, elevation, and physical habitat considered in this study

Attribute	Variable	Abbrev. (unit)	Min	Mean	Max	SD
Chemical water quality	Water temperature	WT (°C)	20.6	25.3	28.8	1.9
	Dissolved oxygen	DO (mg L^−1^)	7.5	8.9	12.0	0.9
	Electric conductivity	EC (μS/cm)	113	286	607	133
	Total suspended solids	TSS (mg L^−1^)	0.9	5.3	17.6	4.7
	Total organic carbon	TOC (mg L^−1^)	1.3	3.4	11.1	2.0
	Biological oxygen demand	BOD (mg L^−1^)	0.4	1.5	4.5	1.1
	Total nitrogen	TN (mg L^−1^)	1.0	2.1	6.0	1.0
	Ammonium-nitrogen	NH_4_-N (mg L^−1^)	0.01	0.08	1.03	0.17
	Nitrate-nitrogen	NO_3_-N (mg L^−1^)	0.6	1.5	4.6	0.8
	Total phosphorus	TP (μg L^−1^)	6.7	57.5	208	42.9
	Orthophosphate	PO_4_-P (μg L^−1^)	0.0	20.4	70.7	18.6
	TN:TP ratio	TN:TP	13.3	66.2	222	59.5
	Sestonic chlorophyll-*a*	Chl-*a* (μg L^−1^)	0.5	18.9	143	32.5
Elevation		Elev (m)	13.0	101	363	77.8
Physical habitat	Stream order	SO	2nd to 6th			
	% of agricultural cover	%Agr	0–49.1			
	% of urban cover	%Urb	1.2–77.8			
	% of forest cover	%For	0–59.2			

*Abbrev*., abbreviation; *Min*, minimum; *Max*, maximum; *SD*, standard deviation

### Hierarchical clustering of chemical water quality conditions

**Fig. 2:**
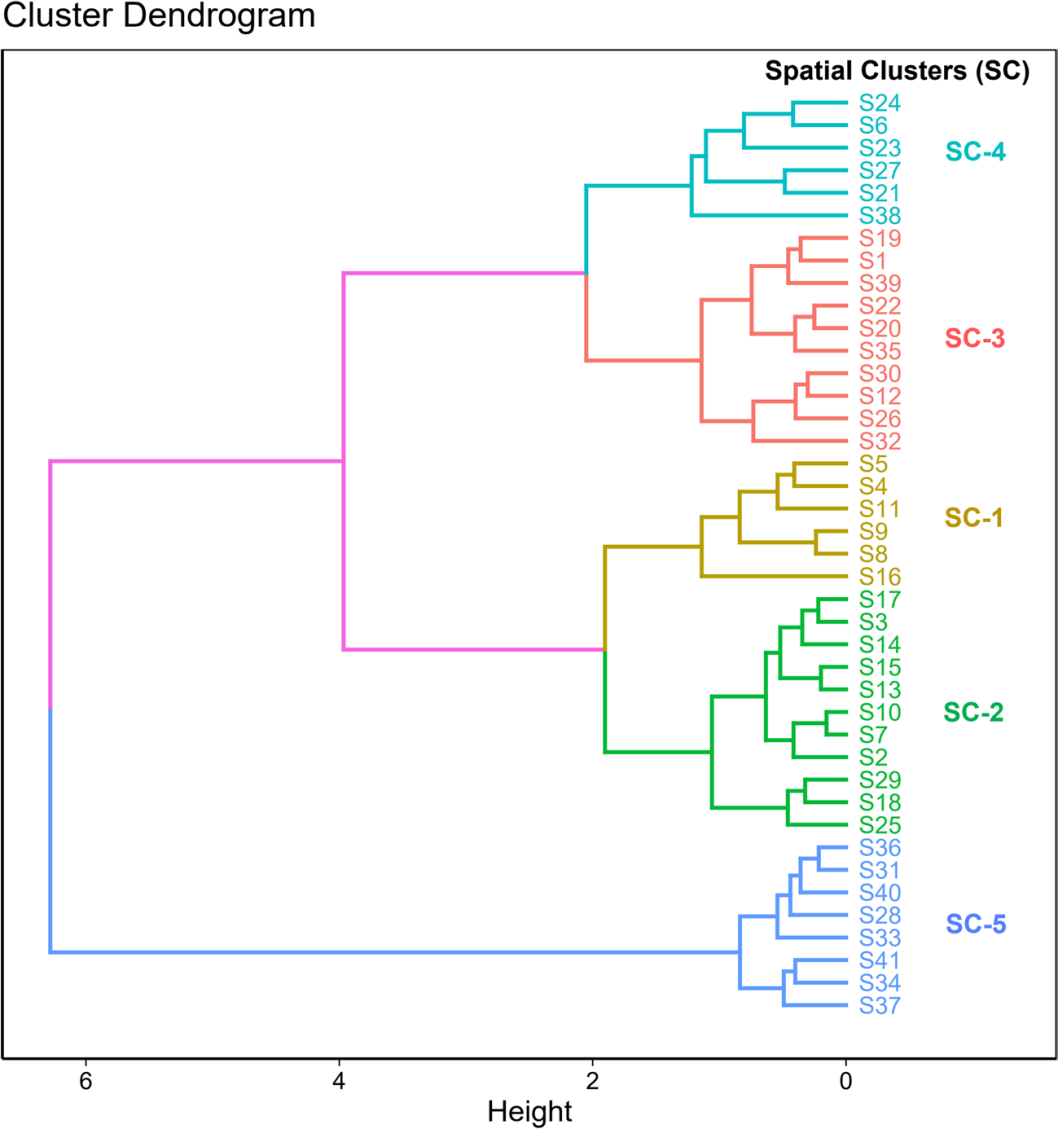
Spatial clusters (SC-1 to SC-5) of water quality based on log-transformed values of water chemistry variables, obtained through hierarchical clustering

We identified five distinct spatial clusters (SCs) that differentiated among sites through hierarchical clustering analysis (Fig. 2). Differences in chemical variables and sestonic Chl-*a* are shown in Table 2.

**Table 2:** Average-defined variation in chemical water quality variables across five spatial clusters

Variable (unit)	Spatial clusters of chemical water condition				
	SC-1	SC-2	SC-3	**SC-4**	SC-5
	Mean (Min–Max)				
WT (°C)	25.1 (22.1–28.0)	25.3 (20.6–27.5)	25.2 (23.2–26.9)	26.2 (22.3–28.8)	25.7 (23.8–27.1)
DO (mg L^−1^)	9.3 (8.2–12.0)	9.0 (7.7–10.7)	8.6 (7.5–10.4)	8.8 (7.9–10.4)	8.9 (8.1–9.6)
EC (μS/cm)	196 (117–336)	176 (113–237)	254 (190–307)	406 (237–607)	452 (324–603)
TSS (mg L^−1^)	1.8 (1.2–3.0)	2.4 (0.9–4.1)	3.5 (1.7–5.1)	5.8 (2.7–11.0)	13.9 (10.0–17.6)
TOC (mg L^−1^)	1.6 (1.3–1.9)	2.7 (1.5–4.1)	2.6 (1.9–3.6)	4.9 (2.5–11.1)	5.8 (4.9–9.2)
BOD (mg L^−1^)	0.8 (0.4–1.0)	0.7 (0.4–0.9)	1.1 (0.6–1.7)	2.0 (1.1–1.9)	3.2 (1.9–4.5)
TN (mg L^−1^)	2.4 (1.4–3.6)	1.6 (1.0–2.0)	1.7 (1.0–2.6)	3.3 (1.0–6.0)	2.5 (1.7–3.2)
NH_4_-N (mg L^−1^)	0.03 (0.01–0.06)	0.03 (0.01–0.04)	0.04 (0.01–0.08)	0.23 (0.02–1.03)	0.12 (0.03–0.35)
NO_3_-N (mg L^−1^)	1.8 (1.1–2.7)	1.2 (0.6–1.5)	1.3 (0.7–2.3)	2.3 (0.8–4.6)	1.6 (0.8–2.0)
TP (μg L^−1^)	11.7 (6.7–16.7)	25.9 (19.0–33.0)	55.0 (37.0–93.7)	87.8 (71–129)	117 (79–208)
PO_4_-P (μg L^−1^)	0.17 (0.0–1.0)	9.2 (1.0–17.7)	21.2 (3.7–39.3)	47.6 (25.3–70.7)	28.3 (7.3–52.3)
TN:TP	199 (172–222)	64.3 (37.6–99.1)	35.2 (13.3–71.8)	42.7 (15.5–82.9)	24.1 (15.4–36.6)
Chl-*a* (μg L^−1^)	2.2 (0.5–3.7)	2.4 (1.2–2.9)	4.4 (1.7–7.2)	14.9 (4.2–22.3)	77.0 (34.5–143)

Variable abbreviations are defined in Table 1

*Abbrev*., abbreviation; *Min*, minimum; *Max*, maximum

Substantial increasing gradients were observed for TSS, EC, organic matter (TOC and BOD), TP, and sestonic Chl-*a* from SC-1 to SC-5 (Table 2). Notably, the concentrations of TSS and Chl-*a* increased sharply in SC-5. WT, DO, and nitrogen levels were relatively constant across the SCs. Dissolved nutrients (NH_4_-N, NO_3_-N, and PO_4_-P) observably increased in SC-4, with substantial variation in the PO_4_-P level from SC-1 to SC-5 (Table 2).

### Variation in chemical health status across SCs

**Fig. 3:**
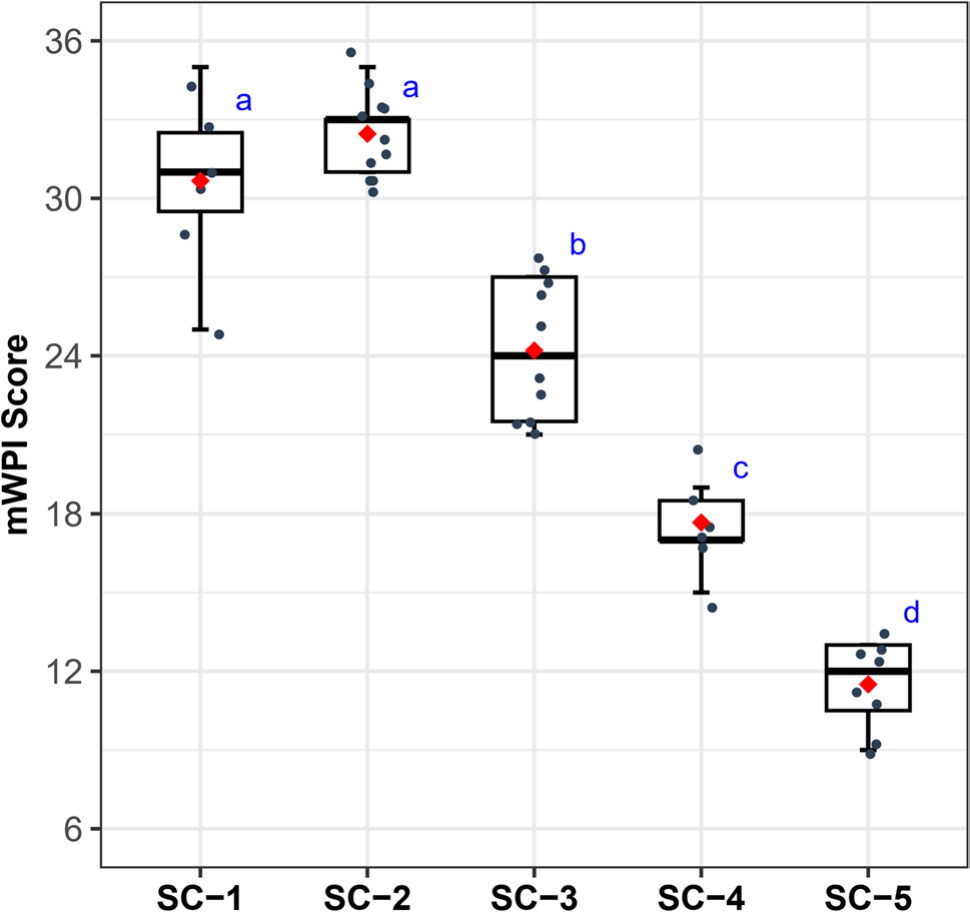
Variation in the multi-metric index of water pollution (mWPI Score) among spatial chemical clusters. Lowercase letters (blue) on each plot indicate significant differences among groups

The mWPI Score significantly decreased from SC-1 and SC-2 to SC-5, which implies chemical health degradation (Fig. 3). SC-1 and SC-2 had mWPI Scores > 24 with averages of 31 and 32, indicating excellent to good health. The mWPI Score distribution in SC-3 ranged between 21 and 27 with a mean of 24, suggesting fair to good health. SC-4 sites had poor to fair health with mWPI Scores ranging from 17 to 21, averaging 18. SC-5 showed very poor health with mWPI Scores ranging from 9 to 13, averaging 11.

### FE-based analysis of stream fish community

We determined 19 FEs based on 50 species identities clustered by their TrG, HG, and TG (Table S4). The number of species within each FE (ranging from 1 to 8) indicated the species richness encompassed by these FEs. Each FE was associated with specific combinations of guilds and abundance distributions across study sites, suggesting the intricate interplay of ecological niches in the study area (Table 3).

**Table 3:** Nineteen fish ecological entities based on trophic (TrG), habitat (HG), and tolerance guilds (TG) and related summary data

Fish ecological entity	Ecological guilds			Number of species	RA of each FE in spatial clusters					Entire		Observed sites (%)
	TrG	HG	TG		SC-1	SC-2	SC-3	SC-4	SC-5	TNI	RA	
FE-1	Omn	WC	IS	8	19.0	11.5	6.5	4.7	4.3	406	8.9	70.7
FE-2	Omn	WC	TS	6	0.3	1.6	1.5	8.8	12.6	217	4.7	48.8
FE-3	Ins	WC	IS	5	5.2	2.8	5.2	2.3	3.6	175	3.8	73.2
FE-4	Omn	BT	TS	4	1.4	0.9	0.3	0.3	0.8	32	0.7	26.8
FE-5	Ins	RB	SS	3	1.6	3.4	1.6	0.7	0.9	82	1.8	36.6
FE-6	Ins	WC	TS	3	0.0	0.5	2.7	7.9	8.3	170	3.7	46.3
FE-7	Ins	RB	IS	3	2.4	12.9	5.7	2.0	4.0	287	6.2	73.2
FE-8	Ins	RB-WC	IS	2	10.1	8.2	3.7	1.2	0.4	219	4.7	65.9
FE-9	Ins	BT	IS	2	1.4	1.8	7.2	8.7	9.4	263	5.7	78.0
FE-10	Car	WC	TS	2	0.3	2.4	2.3	1.5	2.8	93	2.0	51.2
FE-11	Car	BT	TS	2	0.0	0.1	0.0	0.1	0.1	3	0.1	7.3
FE-12	Car	BT	IS	2	0.5	3.4	1.0	0.9	0.9	70	1.5	53.7
FE-13	Car	BT	SS	2	2.1	1.2	0.2	0.3	0.1	33	0.7	31.7
FE-14	Omn	RB	IS	1	6.0	1.2	0.8	0.0	0.2	62	1.3	36.6
FE-15	Omn	RB	SS	1	1.3	3.1	0.3	0.0	0.0	49	1.1	12.2
FE-16	Ins	RB-WC	SS	1	21.3	10.3	8.4	0.0	0.0	353	7.6	34.1
FE-17	Omn	RB-WC	IS	1	27.1	33.9	50.8	59.0	46.9	2022	43.8	100
FE-18	Car	RB-WC	TS	1	0.0	0.2	1.7	1.9	4.5	77	1.7	31.7
FE-19	Ins	BT	TS	1	0.0	0.5	0.2	0.0	0.1	9	0.2	7.3
TNI					621	1213	1147	751	890	4622		*N* = 41
FE-Ric					15	19	18	15	17	19		

TrG: *Omn*, omnivores; *Ins*, insectivores; *Car*, carnivores; HG: *BT*, benthic; *RB*, riffle benthic; *WC*, water column; TG: *SS*, sensitive species; *IS*, intermediate species; *TS*, tolerant species; *TNI*, total number of fish individuals; *RA*, relative abundance; *FE-Rich*, FE-richness; *N*, total study site number

Examining the relative abundances (RAs) of these FEs within each SC revealed spatial patterns in their distribution concerning chemical health conditions (Table 3). For instance, FE-1, consisting of species with the guild identities of Omn, WC, and IS, showed an observable decrease in RA from SC-1 to SC-5, showing a heterogeneous distribution potentially driven by water quality. A significant decrease from SC-1 to SC-5 was also observed in the RAs of FE-8 and FE-16, determined by the Ins, RB-WC, IS, and SS guilds. Notably, FE-16 was absent from sites of SC-4 and SC-5, although it was a dominant FE in the study area. By contrast, the RAs of FE-2 (generalist species with Omn, WC, and TS), FE-9 (species with Car, BT, and TS), and FE-18 (species with Car, RB-WC, and TS) increased spatially from SC-1 to SC-5. Moreover, the exceptional ubiquity of FE-17, an omnivore FE inhabiting the RB-WC and categorized as IS, was notable. This entity was observed in 100% of study sites and was the dominant FE for each SC, which implies its adaptability to diverse chemical and environmental conditions across the study region. Several FEs, including FE-11, FE-15, and FE-19, were present at relatively low rates (< 15%) and RAs (< 1.2%), suggesting their rarity across the study systems (Table 3).

The richness of FEs (FE-Ric) exhibited variation across the chemical SCs, collectively constituting a regional pool of 19 FEs (Table 3). FE-Ric was highest in SC-2 at 19, with a TNI of 1213.

### NMDS ordinations of FEs and mIBI-F metrics

**Fig. 4:**
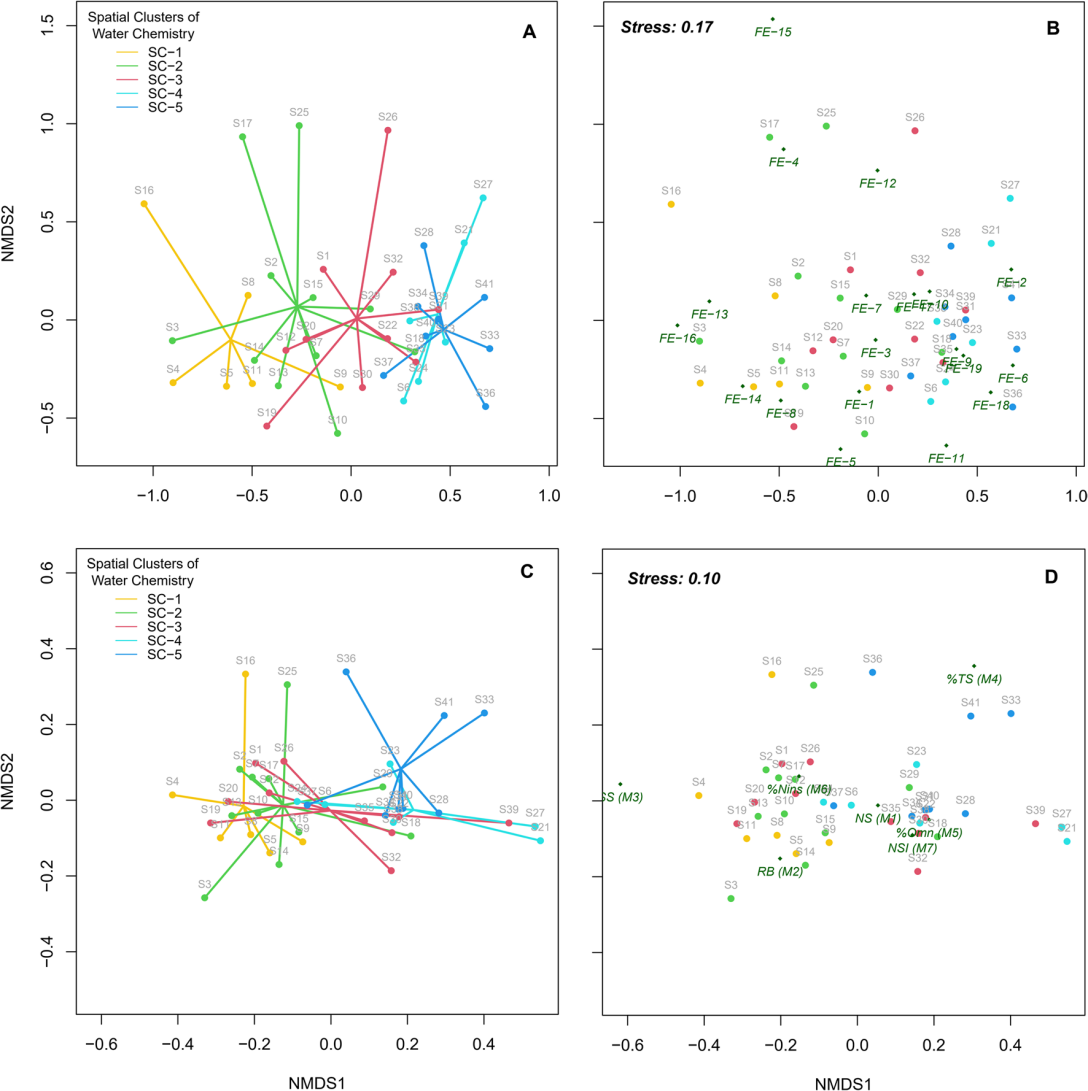
Nonmetric two-dimensional scaling ordination (NMDS1 and NMDS2) depicting fish community composition based on the relative abundance (RA) of fish ecological entities (FEs) (**A** and **B**) and fish-based multi-metric index of biotic integrity (mIBI-F) metrics (**C** and **D**), along with community differences in spatial chemical clusters using spider plots (**A** and **C**). Circles with gray text indicate site scores; circle colors indicate the corresponding spatial chemical clusters. Small diamonds with red text represent FEs and mIBI-F metric scores, respectively

We used NMDS ordination to distinguish differences among fish communities across study sites based on the composition of FEs and mIBI-F metrics. To visualize the results, two-dimensional spider plots were generated using site scores of these community attributes, defined along the NMDS1 and NMDS2 axes (Fig. 4).

Variation in the site scores defined by FEs were generally observed with chemical health degradation from SC-1 to SC-5. The plots also showed a distinct FE-defined score distribution between sites in SC-1 and sites in SC-4 and SC-5. Although mIBI-F metric-defined site scores did not well distinguish the chemical SCs, there were observable differences in site scores between SC-1 and SC-5.

The relative contribution of each FE and mIBI-F metric to site scores along the NMDS1 and NMDS2 axes were analyzed via correlation analysis (Tables S5 and S6). The site scores along the NMSD1 axis of FE had positive correlations with the RAs of FE-2, FE-6, FE-9, and FE-17 but negative correlations with FE-13 and FE-16. The RAs of FE-4, FE-12, and FE-15 were closely associated with the site scores along NMDS2 of FE. For mIBI-F metric-defined axes, except for NS (*M*_1_) and %Ano (*M*_8_), the metrics showed significant correlations with the site scores along the NMDS1, being negatively correlated with the metrics RB (*M*_2_) and SS (*M*_3_) and positively correlated with %Omn (*M*_5_). Meanwhile, the site scores along NMDS2 of mIBI-F showed positive correlations with %TS (*M*_4_) but negative ones with NSI (*M*_7_).

### Spatial response of fish community attributes to chemical health indicators — a regression analysis of ordination site scores

**Fig. 5:**
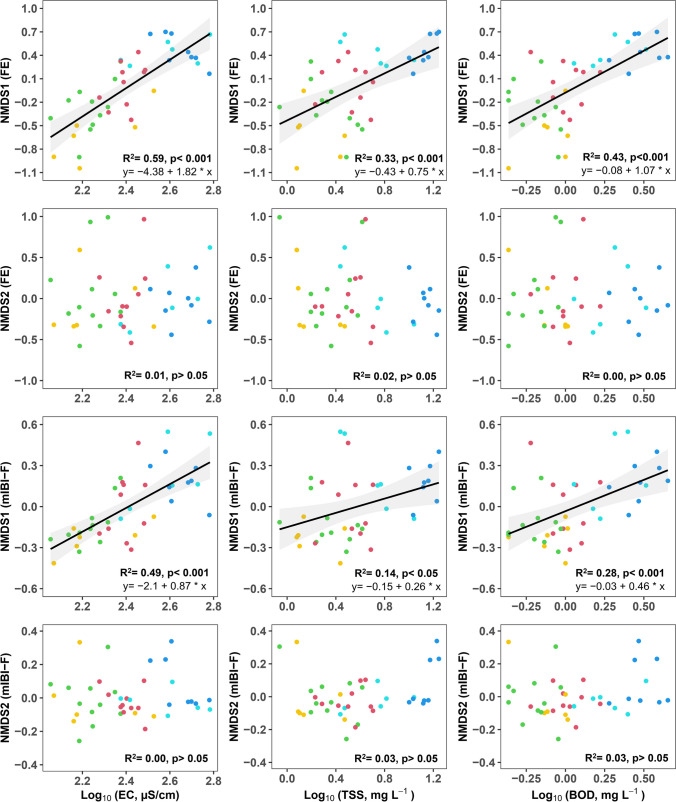
Regression analysis of fish community variation based on FE- and mIBI-F metric-defined scores along the ordinations at study sites, explained by log-transformed electrical conductivity (EC), total suspended solids (TSS), and biological oxygen demand (BOD). Colored circles correspond to the spatial chemical clusters shown in Fig. 2. In each model, black lines indicate the regression lines and shading represents the upper and lower 95% confidence intervals

**Fig. 6:**
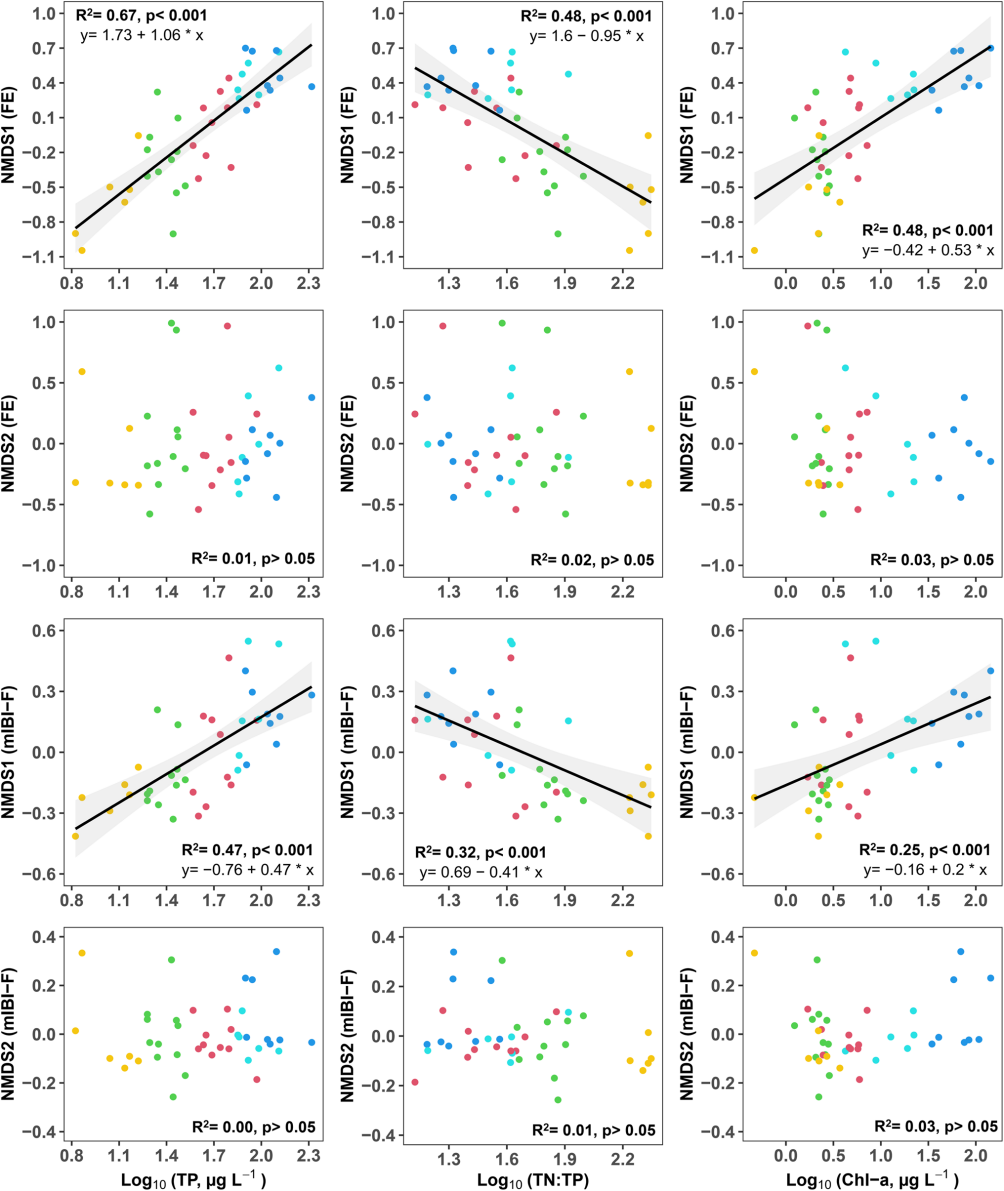
Regression analysis of fish community variation based on FE- and mIBI-F metric-defined scores along the ordinations at study sites, explained by log-transformed total phosphorus (TP), total nitrogen/total phosphorus ratio (TN:TP), and sestonic chlorophyll-*a* (Chl-*a*). Colored circles correspond to the spatial chemical clusters shown in Fig. 2. In each model, black lines indicate the regression lines and shading represents the upper and lower 95% confidence intervals

Regression analysis revealed that chemical health indicators are responsible for spatial shifts in the fish communities described by THE site scores of the NMDS ordinations. Particularly, site scores of NMDS1 (FE) and NMDS1 (mIBI-F) significantly responded to the variation in EC, TSS, BOD, TP, TN:TP, and sestonic Chl-*a* (*p* < 0.001) (Figs. 5 and 6). The explanatory powers of each chemical indicator, measured using the coefficient of determination (*R*^2^), were higher for NMDS1 (FE) than for NMDS1 (mIBI-F). Both these scores were more strongly related to changes in chemical indicators compared to the conventional mIBI-F Score (*R*^2^ < 0.30; Table S7). TP was the most potent variable explaining community variation, followed by EC and Chl-*a*. However, there was no significant linear response of NMDS2 axes to the chemical health indicators (*p* > 0.05).

EC explained 59% and 49% of the variation in site scores along NMDS1 (FE) and NMDS1 (mIBI-F), respectively (Fig. 5). TSS had a significant linear influence on the variation in the scores at study sites along the ordination axes. In addition, BOD, an indicator of organic matter, accounted for 43% and 27% of the variation along NMDS1 (FE) and NMDS1 (mIBI-F), respectively.

As shown in Fig. 6, TP explained 67% and 47% of the variation in site scores along NMDS1 (FE) and NMDS1 (mIBI-F), respectively. In addition, 48% and 32% of site score variation along the respective axes were accounted for by TN:TP ratio. Finally, sestonic Chl-*a* significantly influenced the site score variation along the NMDS1 axes, explaining 48% and 25% of variation in FE and mIBI-F, respectively.

### Correlations among axes, chemical health indicators, and elevation and physical habitat components

**Fig. 7:**
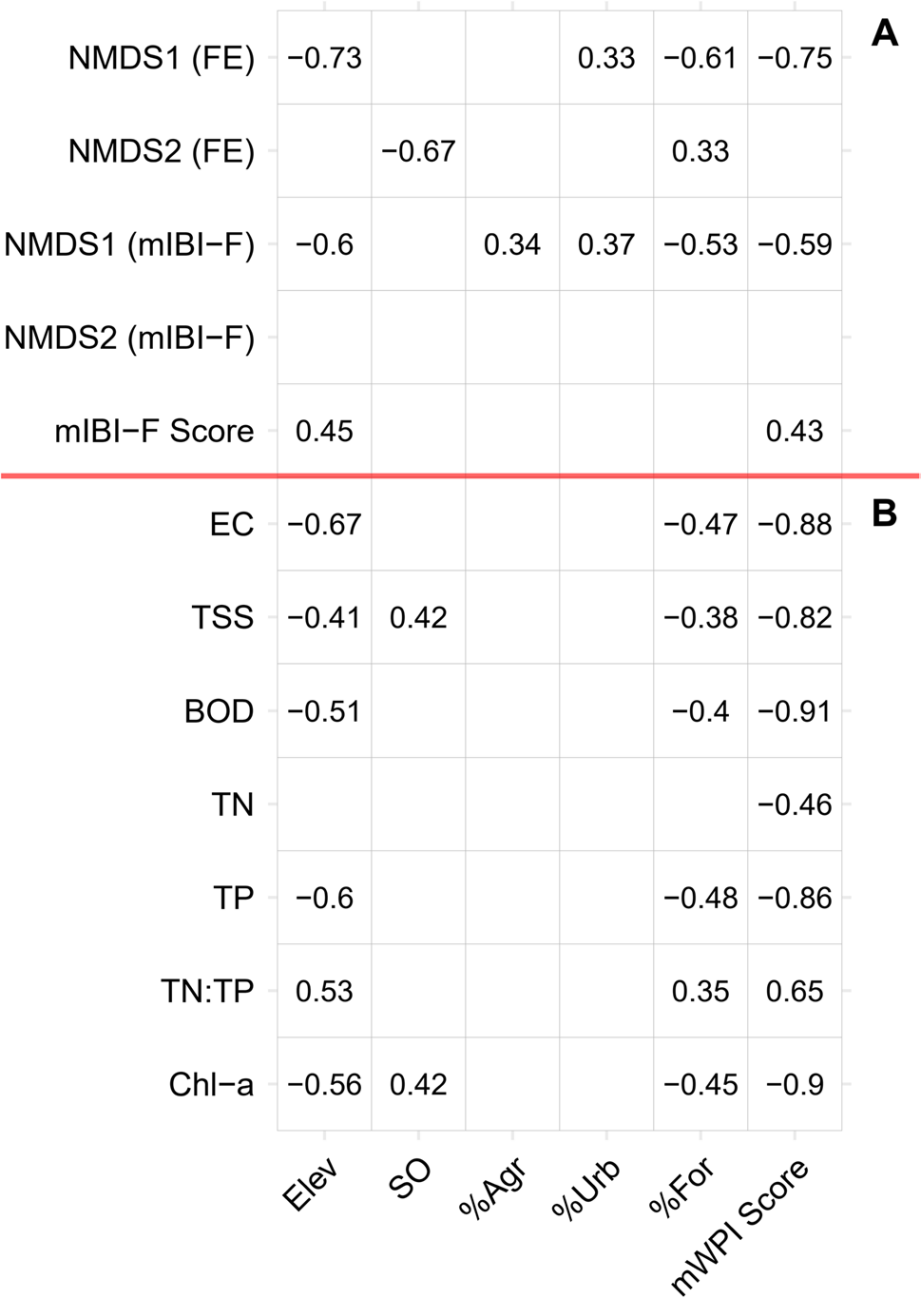
Influences of elevation (Elev), stream order (SO), land use proportion (%Agr, %Urb, and %For), and chemical health scores (mWPI Score) on variation in the fish community defined by ordination scores of FE and mIBI-F, the mIBI-F Score (**A**), and chemical health indicators (**B**). Only significant correlations are shown (Pearson correlation coefficient; *p* < 0.05)

The influences of Elev, SO, riparian land use, and chemical health score (mWPI Score) on variation in the fish community were evaluated using correlation analysis (Fig. 7). Site scores along the NMDS1 axes of FE and mIBI-F were highly responsive to the spatial variation in the environment driven by elevation, riparian land uses, and chemical health. The strongest correlations (*r* > 0.70) were the negative responses of NMDS1 (FE) to Elev and mWPI Score (Fig. 7A). NMDS1 (FE) also showed a strong negative correlation with %For and a moderate positive association with %Urb. NMDS1 (mIBI-F) had strong negative correlations with Elev, %For, and mWPI Score and showed moderate positive correlations with %Urb and %Agr. In addition, NMDS2 (FE) showed a strong negative association with SO and a moderate correlation with %For. There were no significant correlations between NMDS2 (mIBI-F) and the environmental variables. The conventional biological health score (mIBI-F Score) also showed a moderate positive correlation with Elev and mWPI Score (Fig. 7A).

Moreover, most chemical health indicators (except TSS and TN) were strongly associated with Elev and (including TSS) had a moderate correlation with %For (Fig. 7B). These findings indicated that the increase in Elev and %For are associated with a decrease in the concentrations of chemical health indicators across the study region. In addition, TSS and sestonic Chl-*a* were moderately correlated with SO. Finally, there was a substantial contribution (*r* > 0.65) of the chemical health indicators (except for TN) to the mWPI Score.

## Discussion

Deteriorations in water chemistry and physical habitat due to pollution substantially influence the structures of fish communities in both local and spatial groups across wadable rivers and streams (Kalogianni et al. 2017; Whitney et al. 2019; Jargal et al. 2023). Our study provides new insight into how ordination-based site scores of fish community attributes, particularly the composition of FE-based communities, can serve as an effective measure of the ecological health of streams with respect to chemical water quality and changes in riparian land use.

The fundamental premise of biological assessment is that the composition of aquatic communities in disturbed ecosystems is considerably different from that of undisturbed or pristine habitats, commonly referred to as reference sites or groups (Karr 1981; Barbour et al. 1999; Hering et al. 2006). By analyzing these differences, we can obtain valuable insights into a river’s overall health and take necessary measures to protect and preserve it. However, defining reference habitats can be challenging (Ruaro et al. 2020). In this study, we conducted cluster analysis based on water chemistry variables to define reference conditions. The analysis yielded five distinct spatial clusters (SC-1 to SC-5) based on the sites’ chemical conditions, which exhibited clear differences in leading chemical health indicators such as the nutrient regime, suspended, and ionic contents, organic matter, and sestonic Chl-*a* levels. Observable increases in TSS, EC, BOD, TP, and Chl-*a* were found from SC-1 to SC-5, while a decrease in TN:TP was noted. The chemical health was evaluated as excellent to good in SC-1 and SC-2, good to fair in SC-3, fair to poor in SC-4, and very poor in SC-5 based on the mWPI Score. The results demonstrated our reference clusters (SC-1 and SC-2) and those of the disturbed site conditions (SC-4 and SC-5) due to chemical stressors such as high levels of nutrient enrichment, organic matter, and ionic and suspended solids. SC-4 and SC-5 correspond to sites located in the Miho River and Gapcheon Stream, which have been severely impacted by major pollution sources such as wastewater treatment plants, urbanization, and agricultural activities (Yang et al. 2021; Shiferaw et al. 2023).

The different chemical SCs in the study area displayed distinct FE structures based on their richness (FE-Ric) and RA. Although FE-Ric exhibited variation across the SCs, it was not good at indicating the ecological health of water quality in terms of the chemical stressor gradient. However, identifying specific FEs within each SC, particularly those sensitive to environmental changes, can provide valuable insights. Among TGs, sensitive species respond to changes in water quality (Kim et al. 2010; Gao et al. 2015; Whitney et al. 2019), and their presence or absence within certain SCs can indicate the degree of chemical health degradation in those areas. Insectivore fishes are often found in environments that support healthy and diverse insect communities (Barbour et al. 1999; Noble et al. 2007). The RB guild consists of fish species that are well-suited to riffle habitats, where water flows rapidly and is well-oxygenated (Karr 1981; Welcomme et al. 2006).

The FEs defined by these guild identities showed a strong decrease in RA from SC-1 to SC-5, including FE-8 and FE-16. FE-17, which included only one species, *Zacco platypus*, was the sole FE in the dataset observed at 100% of the study sites and which dominated all of the other FEs in each chemical SC, suggesting that FE-17 is not only versatile in terms of its trophic roles and habitat preferences (dual RB-WC preference) but is also highly adaptable to different environmental conditions. By contrast, FE-1 and FE-2 exhibited an increasing RA trend from SC-1 to SC-5. Environmental degradation caused by chemical pollution and land use changes within watersheds is often correlated with a high prevalence of omnivores and tolerant species (Barbour et al. 1999; Ibáñez et al. 2010). The NMDS analysis revealed considerable variation in fish communities between the chemical SCs. This difference was more noticeable between reference and disturbed SCs. Based on our results, environmental filtering, as determined by water chemistry and its relevant chemical stressors, can play a significant role in shaping the feeding and habitat preferences of species found in the studied systems, as well as their tolerance levels. Thus, combining fish species that share identical guild identities into FEs will allow evaluation of the ecological health of wadable rivers and streams using ordination site scores calculated based on weighted FE abundances.

The ordination site scores of FEs and mIBI-F manifested stronger responses to alterations in chemical health indicators and chemical status than conventional mIBI-F scores. The NMDS analysis based on mIBI-F metrics showed observable differences in the fish communities between SC-1 and disturbed chemical SCs. The health indicator metrics in the mIBI-F had a significant correlation (positive or negative) with site scores along the NMDS axes, suggesting the potential for using ordination scores to indicate ecological health status with regard to chemical stressors. The well-defined responses of the metrics to chemical stressor indicators in previous studies support this claim (An et al. 2006; Choi et al. 2011). However, the RA-weighted site scores in NMDS1 of FE showed stronger relations with the chemical indicators (TP, EC, Chl-*a*, and BOD) when compared to scores in NMDS1 of mIBI-F metrics. This suggests that the changes in the structure of FEs determined by ecological guild identities are primarily be attributed to the prevailing water quality conditions. Alterations in fish assemblages are associated with instream chemical conditions driven by nutrient enrichment and high organic matter, leading to a decrease in species abundance and loss of species traits and guild identities, as well as changes in the mIBI-F metrics, due to environmental filtering of chemical stressors (Gao et al. 2015; Kalogianni et al. 2017; Jargal et al. 2023). Additionally, some ecological guilds and mIBI-F metrics associated with generalist feeding preference and tolerance to environmental degradation are positively correlated with chemical stressors (Kim et al. 2010; Atique and An 2018).

The key FEs influencing site variance along NMDS1 were closely associated with the guild indicators of river ecological health, including differences in trophic and tolerance guilds and a preference for riffle habitats. This underscores that using the ordination approach, the alterations in the composition of FEs could provide significant insights into the ecological assessment and monitoring of water quality, as they correlate with the proper functioning of rivers and subsequent variations due to chemical water pollution. Despite the ordination site score of mIBI-F having a weaker correlation with chemical indicators compared to those of FEs, it performed better in capturing the changes in %Agr and %Urb, indicating its usefulness in monitoring ecological health status regarding land use changes. Therefore, the approaches used in the study can complement each other and improve the detection of changes in the ecological health status linked with variations in water chemistry and the resultant chemical stressors and land use effects.

Although river water chemistry plays an important role in shaping local conditions by affecting trait-based sorting of species and community composition, factors such as land use and geographical features can impact the instream components (water chemistry, substrate, and community assembly) (Allan 2004; Kakore et al. 2022; Jargal et al. 2023). In addition to the chemical health score (mWPI Score), Elev and %For had significant influences on variation in the fish community. The site scores along the NMDS1 axes of FE and mIBI-F are sensitive to spatial variation in the environment driven by these factors. The strongest correlations were the negative responses of these axes to mWPI Score, Elev, and %For. Elev and %For were also correlated with variations in chemical health indicators across the study area. Overall, this study highlights the importance of changes in water chemistry along with elevation and decreased %For in shaping the composition of fish communities in wadable rivers and streams. Ordination-based scores provide a valuable tool for using spatial shifts in fish community composition between undisturbed reference environments and chemically disturbed habitats to assess the ecological health of rivers.

## Conclusion

A key challenge in preserving freshwater resources and biodiversity is the presence of chemical pollutants (the widespread issues of nutrient and organic matter enrichment) in rivers and streams. These pollutants are primarily associated with human land use, which severely intensifies with decreased elevation along rivers. While assessments that rely on chemical health indicators and the mWPI are useful for gauging the impact of human activity on freshwater resources, they may not provide a comprehensive understanding of the ecological conditions that impact aquatic organisms. We propose using ordination-based site scores that rely on fish community attributes linked to chemical health indicators to address this issue. Our research demonstrates that such scores can be used to effectively assess the ecological health of river ecosystems in wadable streams, particularly concerning chemical quality conditions and proportional changes in riparian land use along elevation gradients. Specifically, the site scores defined by the NMDS1 axes of FEs and mIBI-F metrics showed better accuracy than the conventional mIBI-F Score for predicting river health. Using fish ordination analysis of the community attributes, we can better determine the impact of chemical health stressors on river ecosystems, identify areas that require attention, and take appropriate measures to preserve the biological integrity of such systems. Additionally, the approach can be used as a baseline for ecological monitoring to assess the effectiveness of management practices over time and make necessary adjustments to management strategies.

### Supplementary Information

Below is the link to the electronic supplementary material.

Supplementary file1 (DOCX 43 KB)

## Data Availability

The data presented in this study may be available on request from the corresponding author.

## References

[CR1] Allan JD (2004) Landscapes and riverscapes: the influence of land use on stream ecosystems. Annu Rev Ecol Evol Syst 35:257–284. 10.1146/annurev.ecolsys.35.120202.110122

[CR2] An KG, Lee JY, Bae DY, Kim JH, Hwang SJ, Won DH, Lee JK, Kim CS (2006). Ecological assessments of aquatic environment using multi-metric model in major nationwide stream watersheds. J Korean Soc Water Environ.

[CR3] Atique U, An KG (2018). Stream health evaluation using a combined approach of multi-metric chemical pollution and biological integrity models. Water.

[CR4] Barbour MT, Gerritsen J, Snyder BD, Stribling JB (1999) Fish protocol. In: Rapid bioassessment protocols for use in streams and wadeable rivers: Periphyton, Benthic Macroinvertebrates and Fish. EPA 841-B-99–002. United states Environmental Protection Agency, Office of Water, Washington, D.C. pp 6–12

[CR5] Bylak A, Kukuła K, Ortyl B, Hałoń E, Demczyk A, Janora-Hołyszko K, Maternia J, Szczurowski Ł, Ziobro J (2022). Small stream catchments in a developing city context: the importance of land cover changes on the ecological status of streams and the possibilities for providing ecosystem services. Sci Total Environ.

[CR6] Bylak A, Kochman-Kędziora N, Kukuła E, Kukuła K (2024). Beaver-related restoration: an opportunity for sandy lowland streams in a human-dominated landscape. J Environ Manag.

[CR7] Carr GM, Neary JP (2008) Water quality for ecosystem and human health, 2nd ed., United nations environment programme global environment monitoring system (GEMS)/Water Programme. Ontario

[CR8] Chalar G, Delbene L, González-Bergonzoni I, Arocena R (2013). Fish assemblage changes along a trophic gradient induced by agricultural activities (Santa Lucía, Uruguay). Ecol Indic.

[CR9] Choi JW, Kumar HK, Han JH, An KG (2011). The development of a regional multimetric fish model based on biological integrity in lotic ecosystems and some factors influencing the stream health. Water Air Soil Pollut.

[CR10] Chua KW, Tan HH, Yeo DC (2019). Loss of endemic fish species drives impacts on functional richness, redundancy and vulnerability in freshwater ecoregions of Sundaland. Biol Conserv.

[CR11] Cortes RMV, Hughes SJ, Pereira VR, Varandas SD (2013). Tools for bioindicator assessment in rivers: the importance of spatial scale, land use patterns and biotic integration. Ecol Indic.

[CR12] Gao X, Zhang Y, Ding S, Zhao R, Meng W (2015). Response of fish communities to environmental changes in an agriculturally dominated watershed (Liao River Basin) in northeastern China. Ecol Eng.

[CR13] Han JH, Park CS, An JW, An KG, Paek WK (2015). Identification guide to freshwater fishes of Korea.

[CR14] Hering D, Feld CK, Moog O, Ofenböck T, Furse MT, Hering D, Brabec K, Buffagni A, Sandin L, Verdonschot PFM (2006). Cook book for the development of a Multimetric Index for biological condition of aquatic ecosystems: experiences from the European AQEM and STAR projects and related initiatives. The Ecological Status of European Rivers: Evaluation and Intercalibration of Assessment Methods. Developments in Hydrobiology.

[CR15] Ibáñez C, Caiola N, Sharpe P, Trobajo R, Jørgensen SE, Xu FL, Costanza R (2010). Ecological indicators to assess the health of river ecosystems. Handbook of Ecological Indicators for Assessment of Ecosystem Health.

[CR16] Jargal N, Atique U, Kim JY, Mamun M, An KG (2022). Functional trait analysis and the multi-metric integrity model, based on stream fish indicators, and their relations to chemical water quality. Water Air Soil Pollut.

[CR17] Jargal N, Mamun M, Choi CY, An KG (2022). Combining functional diversity of lotic fish communities with river health assessment based on multi-metric chemical pollution and biological integrity index models. Front Environ Sci.

[CR18] Jargal N, Kim JE, An KG (2023). New interactive functional indicator approach for river health assessment in an Asian temperate river: comprehensive analysis of water chemistry, physical habitat, land use, and the biological disturbance of invasive alien species. Ecol Indic.

[CR19] Kakore BG, Mamun M, Lee SJ, An KG (2022). Land-use pattern as a key factor determining the water quality, fish guilds, and ecological health in lotic ecosystems of the Asian monsoon region. Water.

[CR20] Kalogianni E, Vourka A, Karaouzas I, Vardakas L, Laschou S, Skoulikidis NT (2017). Combined effects of water stress and pollution on macroinvertebrate and fish assemblages in a Mediterranean intermittent river. Sci Total Environ.

[CR21] Karr JR (1981). Assessment of biotic integrity using fish communities. Fisheries.

[CR22] Keeler BL, Polasky S, Brauman KA, Johnson KA, Finlay JC, O’Neill A, Kovacs K, Dalzell B (2012). Linking water quality and well-being for improved assessment and valuation of ecosystem services. Proc Natl Acad Sci USA.

[CR23] Kim JY, An KG (2015). Integrated ecological river health assessments, based on water chemistry, physical habitat quality and biological integrity. Water.

[CR24] Kim JK, Han JH, An KG (2010). Tolerance range analysis of fish on chemical water quality in aquatic ecosystems. Korean J Ecol Environ.

[CR25] Larentis C, Pavanelli CS, Delariva RL (2021). Do environmental conditions modulated by land use drive fish functional diversity in streams?. Hydrobiologia.

[CR26] Li L, Zheng B, Liu L (2010). Biomonitoring and bioindicators used for river ecosystems: definitions, approaches and trends. Procedia Environ Sci.

[CR27] Mamun M, An KG (2022). Key factors determining water quality, fish community dynamics, and the ecological health in an Asian temperate lotic system. Ecol Inform.

[CR28] McCabe DJ (2011) Rivers and streams: life in flowing water. Nature Education Knowledge 3(10):9. https://www.nature.com/scitable/knowledge/library/rivers-and-streams-life-in-flowing-water-23587918/

[CR29] Mouillot D, Villéger S, Parravicini V, Kulbicki M, Arias-González JE, Bender M, Chabanet P, Floeter SR, Friedlander A, Vigliola L, Bellwood DR (2014). Functional over-redundancy and high functional vulnerability in global fish faunas on tropical reefs. Proc Natl Acad Sci USA.

[CR30] Muñoz I, Sabater S (2014). Integrating chemical and biological status assessment: assembling lines of evidence for the evaluation of river ecosystem risk. Acta Biol Colomb.

[CR31] Nasi F, Vesal SE, Relitti F, Bazzaro M, Teixidó N, Auriemma R, Cibic T (2023). Taxonomic and functional macrofaunal diversity along a gradient of sewage contamination: a three-year study. Environ Pollut.

[CR32] Noble RAA, Cowx IG, Goffaux D, Kestemont P (2007). Assessing the health of European rivers using functional ecological guilds of fish communities: standardising species classification and approaches to metric selection. Fish Manag Ecol.

[CR33] Oksanen J, Blanchet FG, Friendly M, Kindt R, Legendre P, McGlinn D, Minchin PR, O’Hara RB, Simpson GL, Solymos P (2022) Vegan: community ecology package 2.6–2. https://github.com/vegandevs/vegan

[CR34] Oliveira JM, Segurado P, Santos JM, Teixeira A, Ferreira MT, Cortes RV (2012). Modelling stream-fish functional traits in reference conditions: regional and local environmental correlates. PLoS One.

[CR35] Pompeu CR, Penas FJ, Barquín J (2023). Large-scale spatial patterns of riverine communities: niche versus geographical distance. Biodiversity Conserv.

[CR36] Pont D, Hugueny B, Beier U, Goffaux D, Melcher A, Noble R, Rogers C, Roset N, Schmutz S (2006). Assessing river biotic condition at a continental scale: a European approach using functional metrics and fish assemblages. J Appl Ecol.

[CR37] Ruaro R, Gubiani EA, Hughes RM, Mormul RP (2020). Global trends and challenges in multimetric indices of biological condition. Ecol Indic.

[CR38] Shiferaw N, Kim J, Seo D (2023). Identification of pollutant sources and evaluation of water quality improvement alternatives of a large river. Environ Sci Pollut Res.

[CR39] Simon TP, Evans NT (2017) Environmental quality assessment using stream fishes. In: Hauer FR, Lamberti G (Eds.), Methods in Stream Ecology: Vol. 1 Ecosystem Structure. Academic Press, pp 319–334. 10.1016/B978-0-12-813047-6.00017-6

[CR40] Spurgeon J, Pegg M, Parasiewicz P, Rogers J (2019). River-wide habitat availability for fish habitat guilds: implications for in-stream flow protection. Water.

[CR41] Stoddard JL, Herlihy AT, Peck DV, Hughes RM, Whittier TR, Tarquinio E (2008). A process for creating multimetric indices for large-scale aquatic surveys. J North Am Benthol Soc.

[CR42] Vadas RL, Hughes RM, Bae YJ, Baek MJ, Gonzáles OCB, Callisto M, de Carvalho DR, Chen K, Ferreira MT, Fierro P, Harding JS (2022). Assemblage-based biomonitoring of freshwater ecosystem health via multimetric indices: a critical review and suggestions for improving their applicability. Water Biol Secur.

[CR43] Verdonschot PF, van der Lee GH (2020). Perspectives on the functional assessment of multi-stressed stream ecosystems. Freshw Sci.

[CR44] Villéger S, Brosse S, Mouchet M, Mouillot D, Vanni MJ (2017). Functional ecology of fish: current approaches and future challenges. Aquat Sci.

[CR45] Wang C, Jiang Z, Zhou L, Dai B, Song Z (2019). A functional group approach reveals important fish recruitments driven by flood pulses in floodplain ecosystem. Ecol Indic.

[CR46] Welcomme RL, Winemiller KO, Cowx IG (2006). Fish environmental guilds as a tool for assessment of ecological condition of rivers. River Res Appl.

[CR47] Whitney JE, Holloway JA, Scholes DT, King AD (2019). Long-term change of fish communities in a polluted watershed: does cleaner water “act” on fishes?. Trans Am Fish Soc.

[CR48] Yadamsuren O, Morse JC, Hayford B, Gelhaus JK, Adler PH (2020). Macroinvertebrate community responses to land use: a trait-based approach for freshwater biomonitoring in Mongolia. Hydrobiologia.

[CR49] Yang YM, Chae MH, Lee DH, Park YK, Seok KS (2021). Assessment of water quality and sediment pollution in gap stream. J Environ Anal Health Toxicol.

