# Supplementary material for: Ecological river health assessments, based on fish ordination analysis of ecological indicator entities and the biological integrity metrics, responding to the chemical water pollution
Source: Environ Sci Pollut Res Int. 2024 Mar 27;31(19):28306–20. doi: 10.1007/s11356-024-32862-5 (PMC11058779; doi:10.1007/s11356-024-32862-5)
Supplement: Supplementary file 1 — Supplementary file1 (DOCX 43 KB) [file 11356_2024_32862_MOESM1_ESM.docx]

**Supplementary Materials**

**Assessing Ecological Health of Rivers Based on Fish Ordination Analysis of Ecological Indicator Entities and the Biological Integrity Metrics Responding to Chemical Water Pollution**

Namsrai Jargal **^a^**, Jeong-Eun Kim **^a^**^,^ **^b^**, Bilguun Ariunbold **^a^,** Kwang-Guk An **^a,^ ***

**^a^** Department of Bioscience and Biotechnology, Chungnam National University, Daejeon 34134, Republic of Korea

**^b^** Ecological Research Division, Korea National Park Research Institute, Wonju 26411, Republic of Korea

***** Corresponding author: E-mail address: [kgan@cnu.ac.kr](mailto:kgan@cnu.ac.kr) (K-G. An).

**Table S1.** Location of study sites and their stream order and elevations.

| **Study sites** | **Lat** | **Lon** | **Stream and River Name** | **Stream Order** | **Elevation** |
| --- | --- | --- | --- | --- | --- |
| S1 | 35.80211 | 127.5178 | Mainstem [Geum Riv] | 4 | 274 |
| S2 | 35.85632 | 127.4402 | Naeo Stream | 3 | 276 |
| S3 | 35.86685 | 127.6149 | Guryang Stream | 3 | 363 |
| S4 | 35.99068 | 127.6169 | Mainstem [Geum Riv] | 5 | 169 |
| S5 | 36.00304 | 127.6244 | Namdae Stream | 4 | 169 |
| S6 | 36.11059 | 127.5518 | Mainstem [Geum Riv] | 4 | 132 |
| S7 | 36.11106 | 127.5669 | Bonghwang Stream | 5 | 125 |
| S8 | 36.17386 | 127.7744 | Yeongdong Stream | 3 | 124 |
| S9 | 36.2104 | 127.7169 | Yeongdong Stream | 4 | 100 |
| S10 | 36.21207 | 127.7077 | Mainstem [Geum Riv] | 5 | 96 |
| S11 | 36.20561 | 127.9322 | Chogang Stream | 4 | 178 |
| S12 | 36.22049 | 127.9374 | Chupungnyeong Stream | 3 | 175 |
| S13 | 36.2303 | 127.7216 | Chogang Stream | 5 | 97 |
| S14 | 36.28324 | 127.6458 | Mainstem [Geum Riv] | 6 | 87 |
| S15 | 36.27774 | 127.6742 | Mainstem [Geum Riv] | 6 | 74 |
| S16 | 36.54224 | 127.6777 | Bocheong Stream | 2 | 201 |
| S17 | 36.48016 | 127.6881 | Hanggeon Stream | 2 | 165 |
| S18 | 36.48189 | 127.7241 | Bocheong Stream | 3 | 153 |
| S19 | 36.37687 | 127.818 | Bocheong Stream | 5 | 122 |
| S20 | 36.25326 | 127.3252 | Gap Stream | 4 | 73 |
| S21 | 36.33907 | 127.4183 | Daejeon Stream | 3 | 47 |
| S22 | 36.37371 | 127.3794 | Gap Stream | 4 | 36 |
| S23 | 36.4363 | 127.3919 | Gap Stream | 5 | 24 |
| S24 | 36.51375 | 127.3634 | Mainstem [Geum Riv] | 6 | 15 |
| S25 | 36.3421 | 127.4927 | Juwon Stream | 2 | 87 |
| S26 | 36.51356 | 127.5258 | Pongguk Stream | 2 | 83 |
| S27 | 36.97123 | 127.4526 | Seongsan Stream | 3 | 82 |
| S28 | 36.93623 | 127.4616 | Mainstem [Miho Riv] | 3 | 68 |
| S29 | 36.88124 | 127.3799 | Baekguk Stream | 4 | 103 |
| S30 | 36.85398 | 127.4606 | Baekguk Stream | 4 | 60 |
| S31 | 36.84559 | 127.5035 | Mainstem [Miho Riv] | 4 | 52 |
| S32 | 36.78693 | 127.5805 | Bogang Stream | 3 | 55 |
| S33 | 36.77214 | 127.5097 | Mainstem [Miho Riv] | 5 | 41 |
| S34 | 36.71402 | 127.4756 | Mainstem [Miho Riv] | 5 | 34 |
| S35 | 36.63579 | 127.4838 | Musim Stream | 4 | 48 |
| S36 | 36.65838 | 127.383 | Mainstem [Miho Riv] | 5 | 28 |
| S37 | 36.62298 | 127.3507 | Mainstem [Miho Riv] | 5 | 24 |
| S38 | 36.60328 | 127.3058 | Jo Stream | 4 | 27 |
| S39 | 36.57068 | 127.2882 | Wolha Stream | 3 | 26 |
| S40 | 36.52477 | 127.319 | Mainstem [Miho Riv] | 5 | 15 |
| S41 | 36.47798 | 127.271 | Mainstem [Geum Riv] | 6 | 13 |

**Table S2.** The multi-metric model of water pollution index (mWPI) and an integrated chemical indicator with scores assigned based on concentration measurements.

| **Chemical Quality Indicator** | **Model Metric (M)** | **Scoring Criteria** | | |
| --- | --- | --- | --- | --- |
|  |  | **5** | **3** | **1** |
| Nutrient Regime | **M_1_**: Total Nitrogen (TN, mg L^-1^) | < 1.5 | 1.5 - 3.0 | > 3 |
|  | **M_2_**: Total Phosphorus (TP, µg L^-1^) | < 30 | 30 - 100 | > 100 |
|  | **M_3_**: TN:TP Ratio | > 50 | 20 - 50 | <20 |
| Organic Matter Indicator | **M_4_**: Biological oxygen demand (BOD, mg L^-1^) | < 1.0 | 1.0 - 2.5 | > 2.5 |
| Suspended Solid/Ionic Contents | **M_5_**: Total Suspended Solid  (TSS, mg L^-1^) | < 4 | 4 – 10 | > 10 |
|  | **M_6_**: Electric Conductivity  (EC, µS/cm) | < 180 | 180 - 300 | > 300 |
| Primary Productivity | **M_7_**: Sestonic chlorophyll-*a* (CHL-a, µg L^-1^) | < 3 | 3 - 10 | > 10 |
| Chemical Health (Total mWPI Score) | Excellent (31–35), good (25–29), fair (19–23), poor (13–17), and  very poor (07–11) | | | |

**Table S3.** The fish-based multi-metric index of biological integrity (mIBI-F) and an integrated fish community indicator with scores assigned based on corresponding model metrics.

| **Fish Community Indicator** | **Model Metric (M)** | **Scoring Criteria** | | |
| --- | --- | --- | --- | --- |
|  |  | **5** | **3** | **1** |
| Species Richness/ Composition | **M_1_**: Total number of Native  Species | *Expectations of M_1_ - M_3_ vary with stream order* | | |
|  | **M_2_**: Number of Riffle-Benthic Species |  |  |  |
|  | **M_3_**: Number of Sensitive Species |  |  |  |
|  | **M_4_**: Percentage of Individuals as Tolerant Species | < 5 | 5 - 20 | > 20 |
| Trophic Composition | **M_5_**: Percentage of Individuals as Omnivore Species | < 20 | 20 - 45 | > 45 |
|  | **M_6_**: Percentage of Individuals as Native Insectivore Species | > 45 | 45 - 20 | < 20 |
| Fish Abundance and Condition | **M_7_**: Percentage of Total Number of Native Individuals | > 67 | 33 - 67 | < 33 |
|  | **M_8_**: Percentage of Individuals with Anomalies | 0 | 0 - 1 | > 1 |
| Biological Health (Total mIBI-F Score) | Excellent (36–40), good (28–34), fair (20–26), poor (14–18), and very poor (8–13) | | | |

**Table S4.** Fish species recorded across the study sites and their guild identies and relevant fish ecological entities (FE).

| **№** | **Species name (scientific)** | **TrG** | **HG** | **TG** | **FE** |
| --- | --- | --- | --- | --- | --- |
| 1 | *Cyprinus carpio* | Omn | WC | TS | FE-2 |
| 2 | *Carassius auratus* | Omn | WC | TS | FE-2 |
| 3 | *Rhodeus uyekii* | Omn | WC | IS | FE-1 |
| 4 | *Acheilognathus lanceolatus* | Omn | WC | IS | FE-1 |
| 5 | *Acheilognathus koreensis* | Omn | WC | IS | FE-1 |
| 6 | *Acheilognathus yamatsutae* | Omn | WC | IS | FE-1 |
| 7 | *Acheilognathus rhombeus* | Omn | WC | IS | FE-1 |
| 8 | *Acanthorhodeus macropterus* | Omn | WC | IS | FE-1 |
| 9 | *Acanthorhodeus gracilis* | Omn | WC | IS | FE-1 |
| 10 | *Pseudorasbora parva* | Omn | WC | TS | FE-2 |
| 11 | *Pungtungia herzi* | Ins | RB-WC | IS | FE-8 |
| 12 | *Pseudopungtungia nigra* | Ins | RB-WC | IS | FE-8 |
| 13 | *Coreoleuciscus splendidus* | Ins | RB | SS | FE-5 |
| 14 | *Sarcocheilichthys variegatus wakiyae* | Ins | WC | IS | FE-3 |
| 15 | *Sarcocheilichthys nigripinnis morii* | Ins | WC | IS | FE-3 |
| 16 | *Gnathopogon strigatus* | Ins | WC | IS | FE-3 |
| 17 | *Squalidus gracilis majimae* | Ins | WC | IS | FE-3 |
| 18 | *Squalidus japonicus coreanus* | Omn | WC | TS | FE-2 |
| 19 | *Squalidus chankaensis tsuchigae* | Omn | WC | IS | FE-1 |
| 20 | *Hemibarbus labeo* | Ins | WC | TS | FE-6 |
| 21 | *Hemibarbus longirostris* | Ins | WC | IS | FE-3 |
| 22 | *Pseudogobio esocinus* | Ins | BT | IS | FE-9 |
| 23 | *Abbottina springeri* | Omn | BT | TS | FE-4 |
| 24 | *Microphysogobio yaluensis* | Omn | RB | IS | FE-14 |
| 25 | *Microphysogobio jeoni* | Ins | BT | IS | FE-9 |
| 26 | *Rhynchocypris oxycephalus* | Omn | RB | SS | FE-15 |
| 27 | *Zacco koreanus* | Ins | RB-WC | SS | FE-16 |
| 28 | *Zacco platypus* | Omn | RB-WC | IS | FE-17 |
| 29 | *Opsarichthys uncirostris amurensis* | Car | RB-WC | TS | FE-18 |
| 30 | *Erythroculter erythropterus* | Car | WC | TS | FE-10 |
| 31 | *Hemiculter eigenmanni* | Omn | WC | TS | FE-2 |
| 32 | *Misgurnus anguillicaudatus* | Omn | BT | TS | FE-4 |
| 33 | *Misgurnus mizolepis* | Omn | BT | TS | FE-4 |
| 34 | *Iksookimia koreensis* | Ins | RB | IS | FE-7 |
| 35 | *Pseudobagrus fulvidraco* | Ins | BT | TS | FE-19 |
| 36 | *Pseudobagrus koreanus* | Ins | RB | SS | FE-5 |
| 37 | *Silurus asotus* | Car | BT | TS | FE-11 |
| 38 | *Liobagrus andersoni* | Ins | RB | SS | FE-5 |
| 39 | *Oryzias sinensis* | Omn | WC | TS | FE-2 |
| 40 | *Siniperca scherzeri* | Car | BT | IS | FE-12 |
| 41 | *Coreoperca herzi* | Car | BT | SS | FE-13 |
| 42 | *Lepomis macrochirus* | Ins | WC | TS | FE-6 |
| 43 | *Micropterus salmoides* | Car | WC | TS | FE-10 |
| 44 | *Odontobutis platycephala* | Car | BT | SS | FE-13 |
| 45 | *Odontobutis interrupta* | Car | BT | IS | FE-12 |
| 46 | *Rhinogobius brunneus* | Ins | RB | IS | FE-7 |
| 47 | *Rhinogobius giurinus* | Omn | BT | TS | FE-4 |
| 48 | *Tridentiger brevispinis* | Ins | RB | IS | FE-7 |
| 49 | *Macropodus ocellatus* | Ins | WC | TS | FE-6 |
| 50 | *Channa argus* | Car | BT | TS | FE-11 |

**Table S5.** Correlations of fish ecological entities (FEs) to site scores along NMDS1 and NMDS2.

| Fish Ecological  Entities | Two-dimensional scaling ordination | | | |
| --- | --- | --- | --- | --- |
|  | NMDS1 | | NMDS2 | |
|  | r | p-value | r | p-value |
| FE-1 | -0.20 | 0.208 | -0.44 | 0.004 |
| FE-2 | **0.49** | 0.001 | 0.13 | 0.419 |
| FE-3 | -0.04 | 0.805 | -0.17 | 0.301 |
| FE-4 | -0.38 | 0.016 | **0.46** | 0.002 |
| FE-5 | -0.04 | 0.827 | -0.45 | 0.003 |
| FE-6 | **0.58** | 0.000 | -0.23 | 0.143 |
| FE-7 | -0.04 | 0.803 | 0.20 | 0.203 |
| FE-8 | -0.44 | 0.004 | -0.44 | 0.004 |
| FE-9 | **0.56** | 0.000 | -0.33 | 0.035 |
| FE-10 | 0.13 | 0.410 | 0.24 | 0.135 |
| FE-11 | 0.11 | 0.494 | -0.26 | 0.100 |
| FE-12 | -0.20 | 0.210 | **0.74** | 0.000 |
| FE-13 | **-0.58** | 0.000 | 0.08 | 0.631 |
| FE-14 | -0.41 | 0.008 | -0.23 | 0.156 |
| FE-15 | -0.22 | 0.178 | **0.61** | 0.000 |
| FE-16 | **-0.73** | 0.000 | 0.01 | 0.942 |
| FE-17 | **0.57** | 0.000 | 0.21 | 0.193 |
| FE-18 | 0.41 | 0.008 | -0.24 | 0.129 |
| FE-19 | 0.13 | 0.424 | -0.09 | 0.567 |

**Table S6.** Correlations of mIBI-F metrics to site scores along NMDS1 and NMDS2.

| Metrics of mIBI-F | Two-dimensional axes | | | |
| --- | --- | --- | --- | --- |
|  | NMDS1 | | NMDS2 | |
|  | r | p-value | r | p-value |
| NS (M_1_) | -0.04 | 0.819 | -0.27 | 0.094 |
| RB (M_2_) | **-0.54** | 0.000 | -0.33 | 0.036 |
| SS (M_3_) | **-0.78** | 0.000 | -0.02 | 0.910 |
| %TS (M_4_) | **0.46** | 0.003 | **0.65** | 0.000 |
| %Omn (M_5_) | **0.75** | 0.000 | -0.44 | 0.004 |
| %NIns (M_6_) | **-0.81** | 0.000 | 0.16 | 0.323 |
| NSI (M_7_) | **0.46** | 0.003 | **-0.57** | 0.000 |
| %Ano (M_8_) | 0.05 | 0.774 | 0.39 | 0.012 |

**Table S7. The variation in the conventional mIBI-F score (n=41) that explained by log-transformed chemical health indicators.**

|  | **EC** | **TSS** | **BOD** | **TP** | **TN:TP** | **CHL-a** |
| --- | --- | --- | --- | --- | --- | --- |
| Slope | - | - | - | - | + | - |
| R^2^ | 0.15 | 0.19 | 0.16 | 0.30 | 0.22 | 0.23 |
| F-value | 6.73 | 9.31 | 7.93 | 16.73 | 10.93 | 11.33 |
| p-value | <0.05 | <0.01 | <0.01 | <0.001 | 0.002 | 0.002 |
